# The electronic properties of functionalized MXene M_2_XT_2_ (M = Ti, Zr, Sc; X = C; T = O, F) nanoribbon/striped borophene nanoribbon heterojunctions

**DOI:** 10.1039/d4na00629a

**Published:** 2025-05-02

**Authors:** Mahdi Shirazinia, Edris Faizabadi

**Affiliations:** a School of Physics, Iran University of Science and Technology Tehran Iran Mahdishirazi120674@gmail.com edris@iust.ac.ir

## Abstract

The van der Waals heterojunctions and heterostructures developed from diverse materials demonstrate unparalleled potential by combining the favorable properties of their structural layers. In this investigation, we initially showcase the findings and evaluations derived from Density Functional Theory (DFT) of selected functionalized MXene nanoribbons (Ti_2_CO_2_, Zr_2_CO_2_, and Sc_2_CF_2_), along with four types of striped borophene nanoribbons. Nanoribbons come in two forms (armchair and zigzag) and have a variety of widths. Except for 9-, 12-, and 15-MZNRs, there are no band gaps on MXene nanoribbons arranged in a zigzag pattern. Contrastingly, band gaps emerge in MXene nanoribbons with armchair-shaped edges. It is also discovered that every selected SBNR is metallic in nature. Lastly, we carried out a computational analysis of the electronic characteristics of the MNR/SBNR heterojunctions. The significant thermodynamic stability of MNR/SBNR heterojunctions is suggested by the small lattice mismatch in the periodic direction and the negative formation energies. Our research demonstrates that all heterojunction samples exhibit metallic behavior. Additionally, we observed significant changes in total magnetization when applying electric fields of different directions and amplitudes to the heterojunction samples. These findings present promising avenues for enhancing and controlling multiferroics or electrically controllable antiferromagnets, as well as advancing spintronic devices. Moreover, they hold potential for memory devices and sensors.

## Abstract 2

The van der Waals heterojunctions and heterostructures developed from diverse materials demonstrate unparalleled potential by combining the favorable properties of their structural layers.

## Graphical abstract

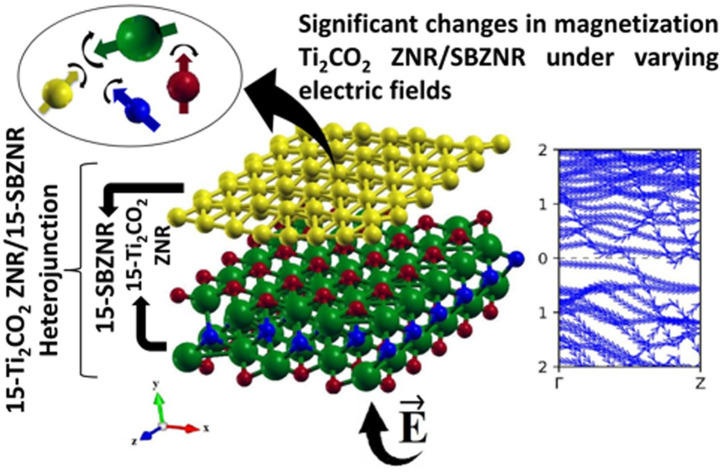

## 1 Introduction

**Fig. 1:**
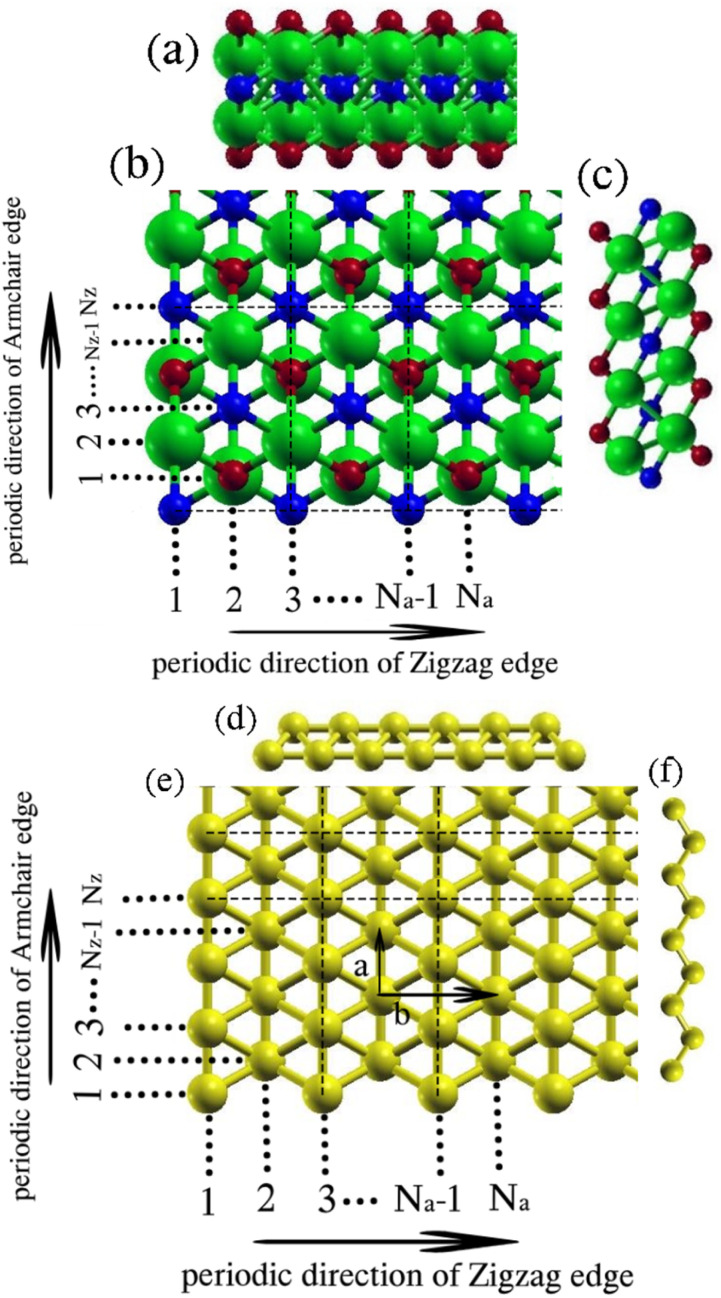
(a), (c), (d) and (f) side views and (b) and (e) top views of M_2_XT_2_ nanoribbons and striped borophene nanoribbons, respectively. M, X, T, and B elements are depicted in green, blue, red, and gold colors, respectively.

The notable characteristics and potential technological applications of two-dimensional (2D) materials and their heterojunctions have garnered significant attention in the realm of nanoscale devices. Semiconducting heterojunctions, in particular, have received a lot of attention due to their intriguing electronic structures and optical characteristics, which have shown promise in photocatalysis, photovoltaics, and optoelectronics. Confining 2D materials leads to the formation of nanoribbons, resulting in significantly different physical characteristics due to surface effects and quantum confinement compared to their 2D counterparts.^1–3^ For instance, graphene nanoribbons, derived from cutting 2D graphene sheets, exhibit diverse electronic and magnetic properties influenced by the chirality of their edge structures.^1,4,5^ Following the discovery of GN, there has been intense interest in employing modern experimental and computational approaches to investigate the properties of elemental 2D materials such as phosphorene,^6,7^ silicone,^8,9^ transition metal dichalcogenides,^10,11^ and germanene.^12,13^ Importantly, the scientific world has been more interested in borophene, a two-dimensional boron sheet, due to its intriguing characteristics, including its high electrical conductivity, distinct electronic features, and preferred surface reactivity.^14–18^ Furthermore, specific arrangements of borophene structures are expected to contain Dirac fermions.^19^ Borophene's applications are expanded to include uses in hydrogen storage, hydrogen detection, and lithium-ion batteries.^20–22^ In practical experiments, on an Ag(111) surface, borophene was effectively grown under extremely high vacuum.^15,17^ Different phases of borophene were observed by high-resolution scanning tunneling microscopy (STM), including striped borophene (SB), β_12_, and χ_3_ phases.^15^ As previously established, the β_12_ and χ_3_ phases of borophene exhibited greater stability compared to the SB phase. This was attributed to their architecture, with 1/6 and 1/5 voids, respectively.^23–29^ Notably, because of its special structure, the SB phase showed characteristic anisotropic metallic nature.^15,16^ Due to this anisotropic nature, the SB phase was observed to possess greater stiffness compared to GN along a direction (as shown in Fig. 1(e)).^15,30,31^

Another type of 2D material that has received substantial attention since its initial introduction by Gogotsi *et al.*^32^ is MXenes. Exfoliation from the MAX phase is a common method for synthesizing these materials.^33^ The MAX phase constitutes an extensive family of triple-layered nitrides, carbonitrides, and transition metal carbides, comprising over 60 members in all. These compounds belong to the space group *p*63/*mmc* and are characterized by the general formula M_*n*+1_AX_*n*_ (where *n* = 1, 2, 3). Here, M denotes an early transition metal, A represents an A-group element (primarily from groups IIIA and IVA), and X signifies either carbon or nitrogen.^34^ The generation of 2D MXenes with the formula M_*n*+1_X_*n*_ (where *n* = 1, 2, 3) can be achieved by eliminating the A group layer from the MAX phase.^32^ MXenes have garnered substantial attention due to their exceptional properties, including resistance to oxidation, electrical conductivity, and impressive damage tolerance.^35,36^ Additionally, MXenes are consistently chemically functionalized with specific groups, especially F and O.^32^ Hence, unaltered MXenes are represented by the formula M_*n*+1_X_*n*_, whereas functionalized MXenes can be denoted by the formula M_*n*+1_X_*n*_T_2_, where T symbolizes functional groups. While many MXenes exhibit metallic properties, their electronic structures can be altered through surface terminations.^37,38^ For instance, computations have shown that Sc_2_CX_2_ (X = F, OH, and O), Ti_2_CO_2_, Zr_2_CO_2_, and Hf_2_CO_2_ display semiconductor behavior.^39^ Earlier investigations additionally suggest that most functionalized MXene systems lack magnetic properties. In contrast, among bare MXenes, only monolayers of Ti_*n*+1_X_*n*_ (X = C, N) exhibit magnetism.^38^ These findings underscore the significant influence of functionalized structure on the characteristics of MXenes.

To date, several first-principles studies on distinct MXene nanoribbons have been published. Zhao *et al.* investigated the electronic characteristics of Ti_2_C, Ti_3_C_2_, and V_2_C nanoribbons in both bare and O-functionalized states at varied sizes.^40^ Zhang *et al.* investigated the carrier mobility of Ti_2_Co_2_ nanoribbons.^41^ Furthermore, Hong *et al.* presented comprehensive findings and studies on many different kinds of terminated MXene nanoribbons derived from 2D monolayers with the composition M_2_XT_2_.^42^ In this study, our initial focus is on presenting the results and analyses of electronic properties for specific functionalized MXene nanoribbons, denoted as M_2_XT_2_ (where M includes Ti, Zr, or Sc; X represents C; T represents O or F), briefly referred to as MNRs (functionalized MXene nanoribbons). The selection of these compounds is based on two fundamental reasons: the first reason is the synthesis and the capability of synthesizing these MXenes. Previous studies and experiments have shown the synthesis of Ti_2_C MXene in the laboratory,^43^ and some studies have also proven the capability of synthesizing Sc_2_C and Zr_2_C.^44^ The second reason is that to examine the structural stability of these MXene nanoribbons, studies have been conducted on their phonon frequencies, and no unstable phonon modes have been observed, which indicates the structural stability of the selected MXene nanoribbons.^42^ Additionally, we explore four variants of striped borophene nanoribbons, briefly referred to as SBNRs. Subsequently, we engage in computational investigations concerning the electronic properties of vertical heterojunctions formed by MNRs and SBNRs. The subsequent sections of the paper are organized as follows: Section 2 provides a brief discussion on models and Section 3 states the technical details underlying our first-principles calculations, while Section 4 begins with the presentation of electronic properties of nanoribbons (MNRs and SBNRs), concluding with an examination of MNR/SBNR heterojunctions.

## 2 Model construction

Previous research has identified three distinct configurations for 2D MXenes M_2_XT_2_. The T atoms in the bottom layer are positioned right under the M atoms in the upper layer in configuration I, and the T atoms in the top layer are positioned above the M atoms in the lower layer in that configuration. The top-layer (bottom-layer) T atoms in configuration II are positioned above (below) the X atoms. With the T atoms in the lower layer positioned under the X atoms in the upper layer and the T atoms in the upper layer positioned right above the M atoms in the bottom layer, configuration III merges configurations I and II. Previous studies have shown that configuration 1 in selected 2D M_2_XT_2_ has the lowest energy structure, implying that it is more stable than other models.^42^ The argument for this is that M atoms (in this case, Ti, Zr, and Sc) supply enough electrons for the X atoms and the T atoms. If M atoms cannot supply enough electrons, model III is the preferred model, where the T atoms can hybridize with the X atoms to obtain the required electrons.^39,45^ Thus, this study uses configuration I. Cutting a ribbon from the 2D sheet in the orthogonal direction yields zigzag or armchair-edge nanoribbons when 1D nanoribbons are constructed. The resulting nanoribbons with zigzag (armchair) edges are characterized by size parameters *n*_z_ (*n*_a_), respectively, as depicted in Fig. 1, and are denoted as *n*_z_-ZNR (*n*_a_-ANR), respectively. In the case of armchair nanoribbons (ANRs), two distinct structural types exist. ANRs with an odd-size parameter have symmetric edges. On the other hand, ANRs with an even-size parameter, have asymmetric edge configurations. The reason for this is that in symmetric ANRs, due to having an odd width, a symmetry line can be found, which is an atomic line parallel to the periodic direction and runs through the center of the nanoribbon. However, in asymmetric ANRs, such an atomic line cannot be found. The classification of Zigzag Nanoribbons (ZNRs) presents a challenge due to the presence of two different types of atomic lines that extend periodically. These atomic lines are made up of rows of X atoms or rows of M and T atoms (where M atoms are atop T atoms or the other way around, generally referred to as M). By cutting the corresponding two-dimensional material into various sizes with zigzag edges, we observe six different edge types of varying sizes. The configuration of atomic lines in ZNRs may be written as ⋯MMXMMX⋯, which results in three different kinds of edges: MMX, MXM, and XMM, where the outer atomic line is indicated by the first letter. As reported in previous studies,^42,46^ there are six different kinds of ZNR structures that may be created when the beginning and conclusion edges are taken into account: (1) *n*_z_-(MMX–MMX)-ZNR, where *n*_z_ = 3*k* (*k* is a positive integer); (2) *n*_z_-(MXM–MXM)-ZNR, where *n*_z_ = 3*k*; (3) *n*_z_-(MMX–MXM)-ZNR, where *n*_z_ = 3*k* + 1; (4) *n*_z_-(XMM–MMX)-ZNR, where *n*_z_ = 3*k* + 1; (5) *n*_*z*_-(MMX–XMM)-ZNR, where *n*_z_ = 3*k* + 2; (6) *n*_z_-(MXM–MMX)-ZNR, where *n*_z_ = 3*k* + 2. Previous studies have shown that the MMX–MMX, MMX–MXM, and MMX–XMM structures have the highest binding energies, indicating that they are more stable than the other structures.^40^ Therefore, in this work, these three structures for ZNRs are considered. The lower edge of the nanoribbons is set as MMX, and the three upper edges are MMX, MXM, and XMM. These configurations allow for the construction of all sizes with these edges. To achieve optimal MNR/SBNR heterojunctions, MNRs are chosen as the substrate, and two models are established, as fully explained in Section 4.2.

## 3 Computational methods

All calculations are conducted utilizing Density Functional Theory (DFT), implemented through the Quantum Espresso package.^47–49^ All of the visualizations are done using the free software, XCrySDen.^50^ The study utilizes the Perdew–Burke–Ernzerhof (PBE) exchange-correlation functional^51^ and the standard solid-state pseudopotentials (SSSP) PBE Precision v1.3.0 for calculations on the Materials Cloud.^52^ We note that previous studies have established the superior accuracy of HSE, mBJ, and HLE potentials for band gap calculations.^53^ Due to computational limitations, additional simulations using HSE, mBJ, and HLE potentials could not be performed in the present study. Nevertheless, it is important to note that QE employs a screening parameter for HSE-like hybrid functionals, and this parameter is utilized at a value of 0.106 (according to Quantum ESPRESSO criteria) in the present manuscript to achieve enhanced accuracy.^54^ Additionally, HSE and PBE functionals have been shown to achieve the mean absolute error (MAE) of 0.687 eV and 1.184 eV, respectively, when compared to experimental band gap values.^55^ Therefore, the MAE of the PBE functional can be considered applicable to all the band gap values reported in this study. Also, some research has reported that the GW band-gap corrections for Ti_2_CO_2_ can reach up to 1 eV.^42^

The convergence threshold on forces for ionic minimization is set to 10^−3^ atomic units (a.u.) as per Quantum ESPRESSO standards. In the case of MNR/SBNR heterojunctions, the calculation incorporates the DFT-D3 version Grimme-D3 (zero damping) to accurately consider van der Waals interactions.^56^ For the MNR/SBNR heterojunction and NR (MNR and SBNR) calculations, plane wave kinetic energy cutoffs of 35 Ry and 45 Ry are applied for the wave functions, and 350 Ry and 450 Ry are employed for the charge density, respectively. Additionally, spin-polarized calculations employ a random initial magnetization. We apply the one-dimensional periodic boundary condition along the nanoribbon's growth direction (*z*) to simulate an infinitely long system. To prevent interactions from periodic images, nanoribbons are spaced 20 Å apart in the nonperiodic directions (*x* and *y*), creating a large vacuum. Additionally, 12 Å vacuum is used to minimize interactions between neighboring periodic components along the nonperiodic directions in heterojunction models. The Broyden–Fletcher–Goldfarb–Shanno (BFGS) approach is used to relax the NRs in the *z*-direction (periodic direction). The calculations are carried out with energy convergence criteria of 2 × 10^−5^ and 10^−7^ Ry between two consecutive self-consistent steps for MNR/SBNR heterojunctions and NRs, respectively. The Brillouin zone is sampled using a Monkhorst-Pack grid of 1 × 1 × 4 for MNR/SBNR heterojunctions and 1 × 1 × 18 for NRs. Following structural optimization, the density of states and other electronic properties are determined through a more dense grid of 1 × 1 × 8 for MNR/SBNR heterojunctions and 1 × 1 × 36 for NRs.

## 4 Results and discussion

### 4.1. Nanoribbons

In this study, we explore three varieties of functionalized 2D MXenes (Ti_2_CO_2_, Zr_2_CO_2_, and Sc_2_CF_2_) alongside striped borophene, aiming to fabricate their corresponding 1D nanoribbons. As an initial step, we calculate the lattice parameters of these 2D structures. The optimized lattice parameters for 2D Ti_2_CO_2_, Zr_2_CO_2_, and Sc_2_CF_2_ are found to be 3.04 Å, 3.31 Å, and 3.29 Å, respectively. These values align well with previous theoretical findings.^42^ The lattice parameters of striped borophene are represented by the values *a* = 1.62 Å and *b* = 2.87 Å, as illustrated in Fig. 1. Notably, the difference in values between the bottom and top atoms can reach up to 0.89 Å.^57^ As outlined earlier, two kinds of nanoribbons are considered: nanoribbons with zigzag and armchair edges. Due to the observed similarity in electronic properties among nanoribbons with the same type of edge, the electronic properties are further investigated in three subsections: MXene zigzag nanoribbons, MXene armchair nanoribbons, and striped borophene nanoribbons. These findings align well with previous first-principles calculations.^42,58,59^

#### 4.1.1. MXene zigzag nanoribbons

**Fig. 2:**
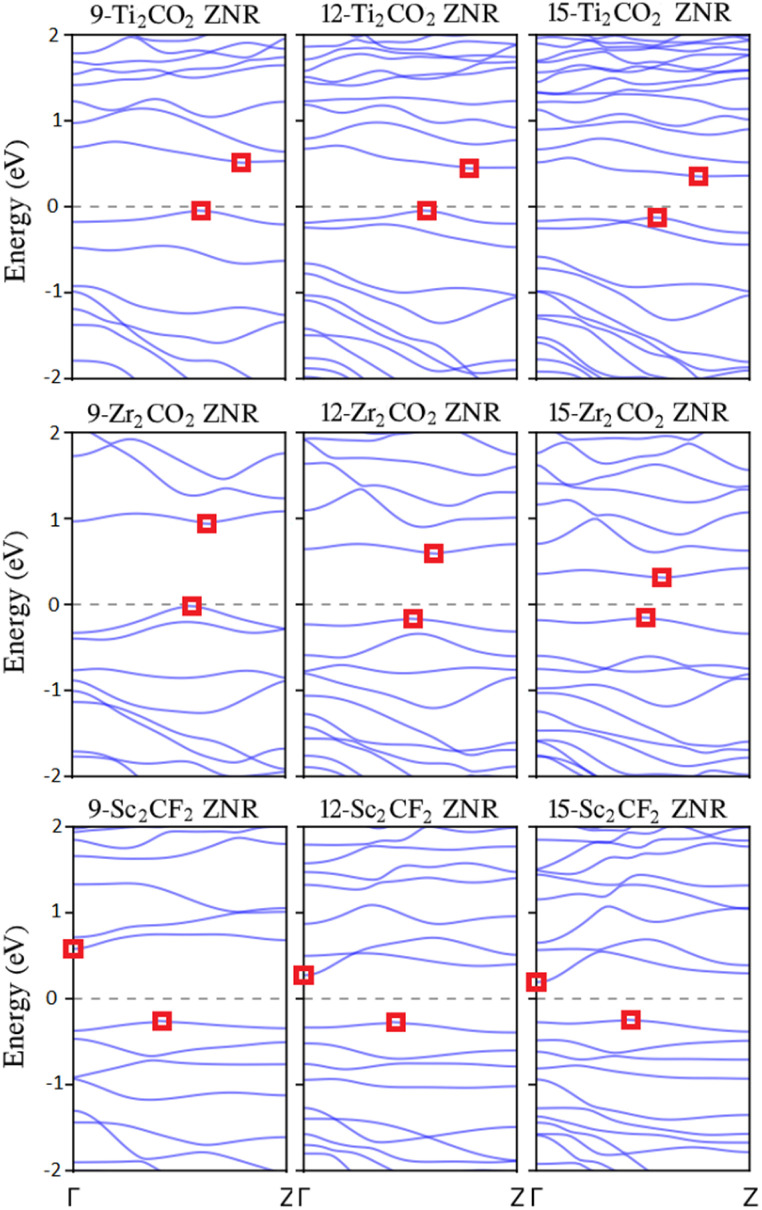
Band structures of semiconducting MZNRs. The Fermi energy is adjusted to zero. The square symbols indicate the positions of the VBM and CBM.

Given that a zigzag nanoribbon edge is defined by the arrangement and sequence of the three outermost atomic lines, it is noteworthy that ZNRs with *n*_z_ < 9 exhibit substantial reconstructions after relaxation. This is attributed to their widths and thicknesses being comparable, resembling more nanorods.^42^ Consequently, for the subsequent analysis, our attention is directed towards ZNRs with sizes in the range *n*_z_ = 9 to 15. According to the descriptions given in Section 2, sizes 9, 12, and 15 have MMX–MMX edges, sizes 10 and 13 have MMX–MXM edges, and sizes 11 and 14 have MMX–XMM edges. Henceforth, for brevity, we will only mention the sizes. The analysis reveals that all the chosen MXene Zigzag Nanoribbons (MZNRs) except 9-ZNR, 12-ZNR, and 15-ZNR exhibit metallic properties. The distinguishing factor among the configurations of 9Z, 12Z, and 15Z from other MZNRs is the presence of X = C elements in the *n*th row of these semiconducting MXenes, as depicted in Fig. 1. In contrast, the metallic MXenes have M elements or a combination of M and T elements in the *n*th row. This elemental composition disparity contributes to the observed semiconductor or metallic character in the respective MXene Zigzag Nanoribbons (MZNRs). Fig. 2 illustrates the band structures of semiconducting zigzag-edged MXenes. The band gaps for 9-ZTi_2_CO_2_, 12-ZTi_2_CO_2_, 15-ZTi_2_CO_2_, 9-ZZr_2_CO_2_, 12-ZZr_2_CO_2_, 15-ZZr_2_CO_2_, 9-ZSc_2_CF_2_, 12-ZSc_2_CF_2_, and 15-ZSc_2_CF_2_ across the energy range are 0.57 eV, 0.50 eV, 0.48 eV, 0.98 eV, 0.77 eV, 0.47 eV, 0.85 eV, 0.55 eV, and 0.44 eV, respectively. The band gaps exhibit a monotonic decrease with increasing width of the nanoribbons. The observed trend is explained by the quantum confinement effect. For further discussion, we note that the band gaps of semiconducting ZNRs have been expected to shift towards their corresponding 2D band gaps as their width increases. As described in Section 1, 2D Ti_2_CO_2_, Zr_2_CO_2_, and Sc_2_CF_2_ are semiconductors with band gaps of 0.32, 0.97, and 1.03, respectively. In the semiconducting Ti_2_CO_2_ ZNRs, the band gaps clearly converge to the band gap of 2D Ti_2_CO_2_, and the computed band gaps in this article converge to 0.32. However, this expectation is not found in the computed band gaps of semiconducting Zr_2_CO_2_ and Sc_2_CF_2_ ZNRs. Previous research on the wave-function properties of the valence and conduction bands of the semiconducting MZNRs has revealed that edge states typically appear at the CBM, and the VBM is bulklike, with full edge states occurring down the valence band.^42^ The existence of edge states can affect the electronic band structure of a material, potentially reducing the band gap. This is because edge states can add new energy levels into the band gap, essentially closing it. For example, in graphene, edge states at zigzag edges can introduce localized states within the band gap, altering the electrical characteristics of the material.^60,61^ In all cases, both the conduction band minimum (CBM) and the valence band maximum (VBM) are positioned between the *Γ* and *Z* points, except for the CBM of semiconducting zigzag-edged Sc_2_CF_2_ nanoribbons, which is located at the *Γ* point. As discussed, this anomaly can indicate the presence of edge states at this point. The presence of these indirect band gaps is crucial for the semiconducting behavior observed in devices incorporating 1D MXene.

**Fig. 3:**
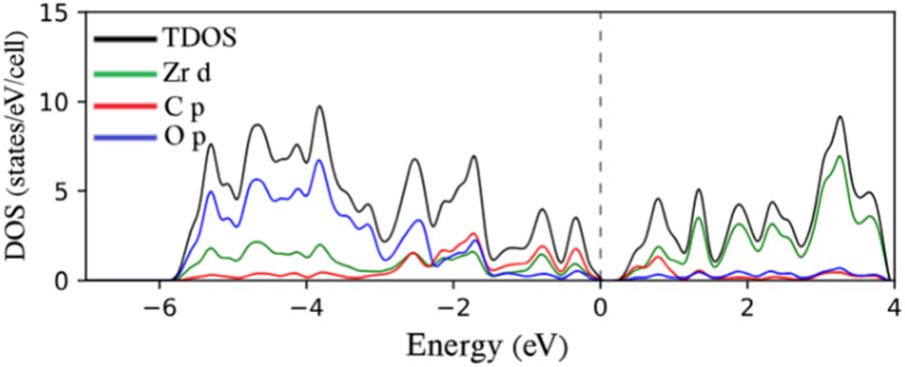
TDOS and PDOS on selected atomic orbitals of 9-Zr_2_CO_2_ ZNR. The Fermi energy is adjusted to zero.

Fig. 3 shows the total density of states (TDOS) and projected density of states (PDOS) for the representative 9-Zr_2_CO_2_ ZNR. In all samples, the conduction band primarily stems from M = Ti, Zr, and Sc d states, while the valence levels between −7 and 0 eV have been partitioned by two subbands. In semiconducting Ti_2_CO_2_ ZNRs and Zr_2_CO_2_ ZNRs, subband I between ∼−3 and 0 eV exhibits nearly equal contributions from M = Ti d, Zr d, C p, and O p (with more C p) orbitals. Subband II, ranging from approximately −6 to −3 eV, is mainly characterized by O p orbitals, with some contribution from M = Ti d and Zr d characteristics because of the strong hybridization between them. For semiconducting SC_2_CF_2_ ZNRs, these subbands are distinctly separated, and two subbands are distinguished by a tiny gap (∼1 eV). Subband I, ranging from ∼−3.5 to 0 eV, displays nearly equal contributions from Sc d, C p (with more C p), while subband II, spanning ∼−7 to −4.6 eV, is primarily dominated by F p orbitals. The significant similarity in the electronic properties of the ZNRs constructed from these three selected functionalized 2D MXenes in this article (which also appears in the next section for ANRs) is due to the fact that the selected elements are very close to each other in the periodic table and all three have the lowest energy structure in Model 1, as discussed in the model section. This result, which was claimed in another study,^42^ is substantiated by the findings of this article.

#### 4.1.2. MXene armchair nanoribbons

**Fig. 4:**
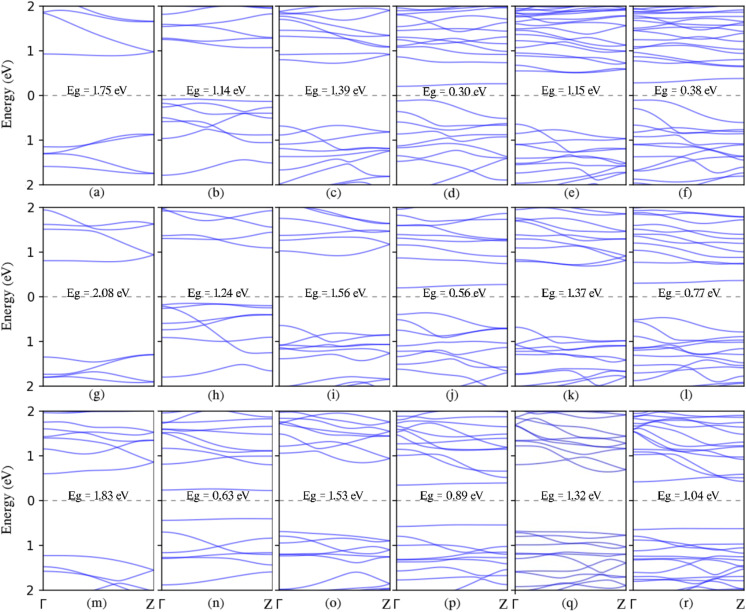
Band structures of all selected MANRs (*n*_a_ = 2–7): (a)–(f) Ti_2_CO_2_ armchair nanoribbons; (g)–(l) Zr_2_CO_2_ armchair nanoribbons; (m)–(r) Sc_2_CF_2_ armchair nanoribbons. The Fermi energy is adjusted to zero, and band gaps are written with their values.

Using the 2D MXene sheet, armchair nanoribbons (ANRs) of M_2_XT_2_ with different widths are built. The interval of the width parameter, *n*_a_, is 2 to 7. Our results show that the semiconducting feature is present in all ANRs. The semiconductor character of armchair nanoribbons has also been observed in various other 2D sheets.^46,62^ The band structure and indirect band gaps of selected 2 A to 7 A M_2_XT_2_ over the energy range are depicted in Fig. 4. As illustrated in the figure, every nanoribbon with asymmetric (even) edges has a larger band gap than the one having symmetric (odd) edges. To examine this phenomenon, we take a look at the structure of unit cells of symmetric and asymmetric MANRs. As shown in Fig. 1(b), the unit cell of asymmetric MANRs with an even width, regardless of X and T elements, consists of two rows of element M, with each row having element M as much as half the width. If one element M is added to or removed from one of the rows, the next or previous width unit cell is obtained. For example, the 4-MANR unit cell has two rows of element M, each row containing two elements of M. If one element M is added to one of the rows, we will have a unit cell with one row of two elements of M and one row of three elements of M, which is the 5-MANR unit cell. If one element M is removed from one of the rows, we will have a unit cell with one row of two elements of M and one element M in the other row, which is the structure of the 3-MANR unit cell. Before relaxation, the distance between M atoms in the unit cell equals the corresponding 2D lattice constant. However, after relaxation, one of the changes in symmetric MANR unit cells is that in the row with more M elements, its M atoms significantly move closer together. For example, the average distance change in 7-Sc_2_CF_2_ ANR is a reduction of 3.95%. This significant reduction in the distance of M elements creates strong d–d hybridization between M (transition metal) elements. Since such significant reduction is not observed in asymmetric MANRs (for example about 1.8% on average in 6-Sc_2_CF_2_ ANR), each nanoribbon with asymmetric edges has a larger bandgap compared to a ribbon with symmetric edges. It is worth mentioning that this distance reduction in symmetric Sc_2_CF_2_ ANR samples significantly increases with reduced width. In the 5-Sc_2_CF_2_ ANR, the reduction is 5.17%, and in the 3-Sc_2_CF_2_ ANR, it is 7.9%. This can be a reason that the band gaps of symmetric Sc_2_CF_2_ ANRs increase with increasing width. These results align with those observed in earlier research.^42,63^ The band gaps of asymmetric edges narrow as their width number increases, a consequence attributed to the conventional quantum confinement effect. Also examining Ti_2_CO_2_ armchair nanoribbons in the figures reveals the presence of edge bands (the first band above the Fermi energy) situated among the valence and conduction bands upon generating NRs from the 2D Ti_2_CO_2_ sheet. This suggests that the presence of electronic states at the edge of symmetric armchair Ti_2_CO_2_ nanoribbons causes a downshift in CBM energy, which lowers the obvious band gap for nanoribbons that are thinner. If we disregard the edge bands, the gaps of the valence and conduction bands appear to narrow as the width number of the NRs increases, a manifestation of the conventional quantum confinement effect. It is worth noting that Hong *et al.* have demonstrated charge density isosurfaces with an isovalue of 0.02 e^−^ Å^−3^ for some edge states within the band structure of 7-Ti_2_CO_2_ ANR;^42^ in addition, these results align with observations reported in previous studies.^58^ Hence, the edge effect of MANRs emerges as a crucial factor influencing their electronic properties. For all samples of 5- and 7-armchair nanoribbons (MANRs), the CBM is located at the *Γ* point, and the VBM is situated between the *Γ* and *Z* points. The CBM and the VBM are located between the *Z* and *Γ* points and at the *Γ* point, respectively, in every case of 6-armchair nanoribbons. In other cases, the CBM and the VBM are positioned at different points in the Brillouin zone.

**Fig. 5:**
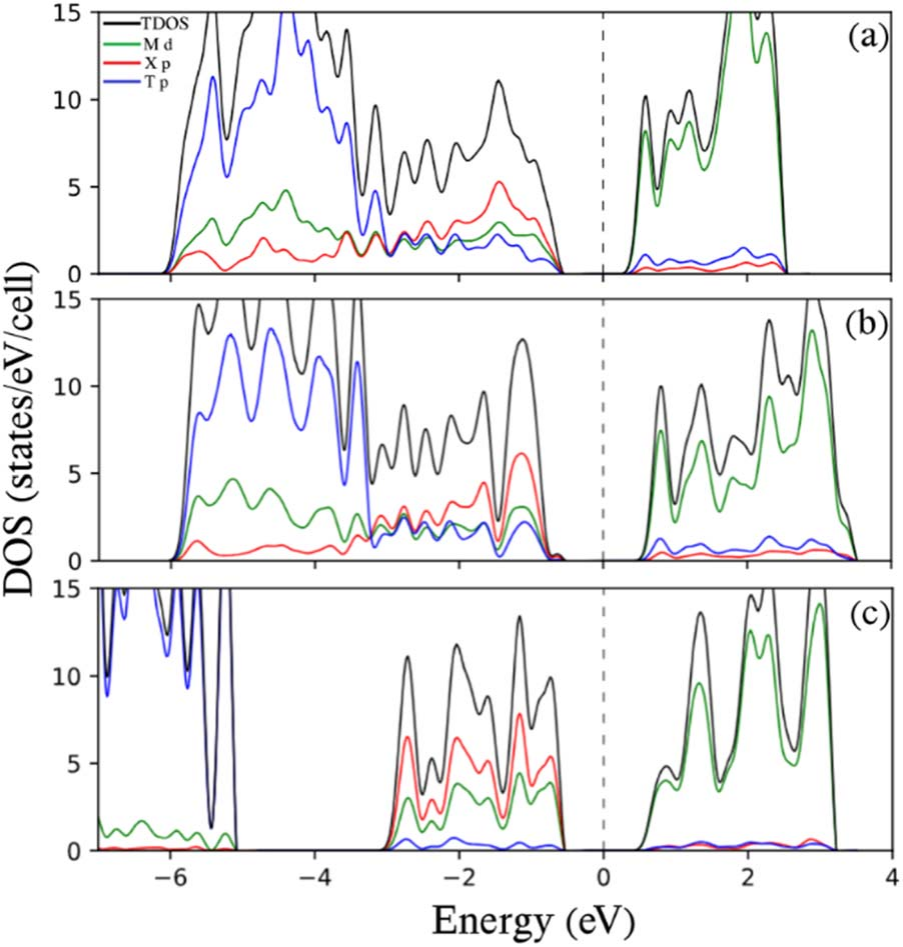
TDOS and PDOS on chosen atomic orbitals of (a) 6-Ti_2_CO_2_ ANR, (b) 6-Zr_2_CO_2_ ANR, and (c) 6-Sc_2_CF_2_ ANR. The Fermi energy is adjusted to zero.

This phenomenon is also evident in the Density of States (DOS) plots depicted in Fig. 5. The analysis of Projected Density of States (PDOS) for armchair-edged M_2_XT_2_ nanoribbons mirrors the PDOS analysis of their zigzag-edged counterparts. In both symmetric and asymmetric samples, the conduction band is predominantly attributed to M = Ti d, Zr d, and Sc d states, while the valence states within the range of −7 to 0 eV can be categorized into two subbands. For semiconducting Ti_2_CO_2_ armchair nanoribbons and Zr_2_CO_2_ armchair nanoribbons, subband I, spanning approximately −3 to 0 eV, exhibits nearly equal contributions from M = Ti d, Zr d, C p, and O p (with more C p) orbitals. Subband II, between approximately −6 and −3 eV, is primarily dominated by O p orbitals, with some M = Ti d and Zr d character (more O p) because of their influential hybridization. In semiconducting SC_2_CF_2_ armchair nanoribbons, these subbands are distinctly separate. Subbands I and II are split by a tiny gap (∼2 eV). Subband I, between approximately −3 and 0 eV, shows almost identical contributions from Sc d and C p (with more C p) orbitals, while Subband II, spanning approximately −7 to −5 eV, is primarily dominated by F p orbitals.

#### 4.1.3. Striped borophene nanoribbons

**Fig. 6:**
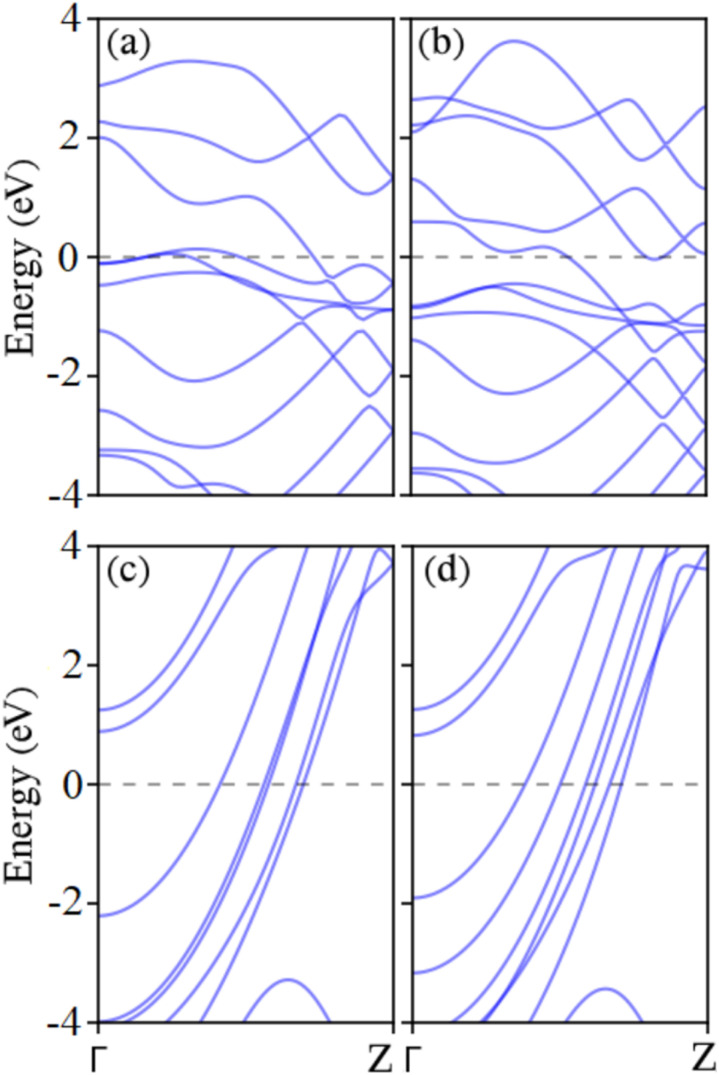
The band structures of (a) 12-ZSBNR, (b) 15-ZSBNR, (c) 6-ASBNR, and (d) 7-ASBNR. The Fermi energy is adjusted to zero.

To facilitate a more comprehensive examination of MNR/SBNR heterojunctions, which will be discussed in the following section, we investigate the electronic properties of four types of striped borophene nanoribbons: 12-ZSBNR, 15-ZSBNR, 6-ASBNR, and 7-ASBNR. These nanoribbons are constructed from the 2D striped borophene sheet, as illustrated in Fig. 1. Fig. 6 presents the band structure of these selected SBNRs. Our findings indicate that these nanoribbons, characterized by different edges and widths, exhibit metallic properties, thereby inheriting the metallic characteristics of striped borophene.^64^

### 4.2. MNR/SBNR heterojunctions

In this section, the electronic properties of MNR/SBNR heterojunctions with the same widths have been examined. The optimized lattice parameters of 2D M_2_XT_2_ and striped borophene are expressed in the first part of Section 4.1. The lattice mismatch ratio can be calculated as *σ* = (*a*_2_ − *a*_1_)/*a*_1_, where *a*_1_ and *a*_2_ represent the lattice parameters of the substrate and film, respectively. To achieve optimal MNR/SBNR heterojunctions, MNRs were chosen as the substrate, and various models were evaluated. The lattice mismatch ratio for each model was calculated, and the model with the smallest lattice mismatch ratio as the superior model was chosen for further analysis. Superior models are as follows: for armchair edges (Model A), a supercell with seven relaxed primitive cells of SBANRs is vertically superimposed on the top of two relaxed primitive cells of MANRs; for zigzag edges (Model B), a supercell with one relaxed primitive cell of SBZNRs is vertically superimposed on the top of one relaxed primitive cell of MZNRs. Other models with larger lattice mismatches were not considered. Table 1 presents the lattice mismatch ratios of Model A and Model B along the periodic direction. The results indicate that the lattice mismatch ratios of Zr_2_CO_2_ ANR/SBANR and Sc_2_CF_2_ ANR/SBANR are sufficiently low, ensuring reliable results for van der Waals (vdW) heterojunctions. However, the creation of Ti_2_CO_2_ ZNR/SBZNR is still anticipated since MXenes can endure very large tensile strains.^65^ Therefore, we have chosen Zr_2_CO_2_ ANR/SBANR, Sc_2_CF_2_ ANR/SBANR, and Ti_2_CO_2_ ZNR/SBZNR as samples for heterojunctions.

**Table 1:** The lattice constants (Å) for NRs and lattice mismatch ratios of Model A and Model B for MNR/SBNR heterojunctions

Models	Lattice constants (this work)				Lattice mismatch ratios		
	Ti_2_CO_2_ NR	Zr_2_CO_2_ NR	Sc_2_CF_2_ NR	SBNR	Ti_2_CO_2_ NR/SBNR	Zr_2_CO_2_ NR/SBNR	Sc_2_CF_2_ NR/SBNR
Model A (armchair edge)	5.26	5.73	5.69	1.62	7.69%	−1.04%	−0.44%
Model B (zigzag edge)	3.04	3.31	3.29	2.87	−5.59%	−13.29%	−12.77%

We specifically considered widths 6 and 7 for armchair nanoribbons and 12 and 15 for zigzag nanoribbons. This choice was driven by two main factors: 1. these specific widths in zigzag nanoribbons exhibit semiconductor behavior, and so we aimed to select semiconductor samples. 2. The observation that nanoribbons with these particular widths demonstrate more negative energy indicates greater stability compared to other widths. In summary, we have a total of six samples for heterojunctions: 6-Sc_2_CF_2_ ANR/6-SBANR, 7-Sc_2_CF_2_ ANR/7-SBANR, 6-Zr_2_CO_2_ ANR/6-SBANR, 7-Zr_2_CO_2_ ANR/7-SBANR, 12-Ti_2_CO_2_ ZNR/12-SBZNR, and 15-Ti_2_CO_2_ ZNR/15-SBZNR. We note that heterojunctions can generally be categorized into three types based on the materials involved: one is a semiconductor heterojunction which involves the interface between two different semiconductor materials. The formation of a potential well at the contact is one advantageous characteristic of the semiconductor heterojunction. Regarding this potential well, in the direction perpendicular to the interface, electrons are stuck; in the other two directions, they are free to travel.^66^ Another type of heterojunction is metal heterojunction which involves the interface between two different metals. The unique capabilities of metal heterojunctions make them appealing nanostructures. For instance, they have been shown to enhance the catalytic activity and electrochemically active surface^67–70^ with recent progress in liquid fuel cells.^71^ The last type of heterojunction – all our samples in this paper are of this type – is the metal–semiconductor junction which involves the interface between a metal and a semiconductor. Metal–semiconductor junctions are crucial due to their distinct architecture, electrical and optical characteristics, and use in optoelectronic devices, particularly in diode manufacturing.^72–75^ The metal–semiconductor diodes were one of the most beneficial developments at the beginning of the nineteenth century. A metallic whisker was touched to an exposing semiconductor surface to create this diode. When a metal is placed onto a lightly doped semiconductor, it will create a rectifying contact known as a Schottky barrier diode. Additionally, this type of heterojunction is able to generate ohmic contacts, which are low-resistance interfaces that allow current to flow in both directions while reducing the voltage drop across the junction.^66^

**Fig. 7:**
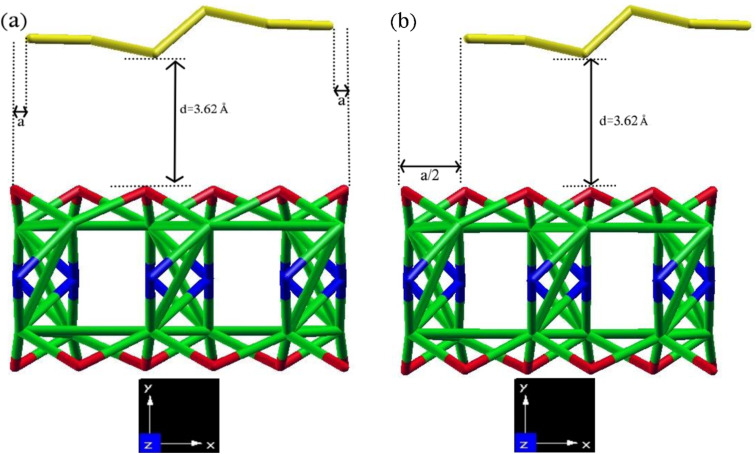
The schematic views of the crystal structure of a representative 6-Zr_2_CO_2_ ANR/6-SBANR heterojunction in stick display mode of (a) configuration I and (b) configuration II. In this specific case, the stick display mode highlights the differences between configurations and distances better than other modes, thereby facilitating a better understanding. The unit cells are relaxed and placed at an interlayer distance of 3.62 Å. *z* is the periodic direction, *a* in configuration II represents the lattice parameters of the 2D Zr_2_CO_2_ MXenes (3.31 Å). Zr, C, O, and B elements are represented by green, blue, red, and gold balls, respectively.

We have considered an equilibrium interlayer distance of 3.62 Å, which is determined by the distance between the minimum *y* value of the relaxed primitive cell of SBNR atoms and the maximum *y* value of the relaxed primitive cell of MNR atoms, as illustrated in Fig. 7. This choice aligns well with previous studies on other van der Waals (vdW) heterojunctions.^76,77^ To elucidate the effects of different alignments between the two layers, we considered two configurations, as depicted in Fig. 7: (1) SBNRs are positioned in the middle of the *xy*-plane of MNRs, maintaining the same distance ‘*a*’ from the top and bottom. (2) The SBNRs are shifted by half of the lattice parameters of the corresponding 2D selected MXenes from the bottom. In this configuration, the bottom edge is situated inside the *xy*-plane of MNRs, while the upper edge extends beyond the *xy*-plane of MNRs. It is observed that configuration I exhibited more negative energy in all samples (except for 6-Sc_2_CF_2_ ANR/6-SBANR), indicating superior performance compared to configuration II. Consequently, we chose configuration II for 6-Sc_2_CF_2_ ANR/6-SBANR and configuration I for other heterojunctions.

To evaluate the thermodynamic stability of MNR/SBNR heterojunctions, their formation energy (*E*_f_) is calculated using the following equation:*E*_f_ = *E*_MNR/SBNR heterojunction_ − *E*_MNR_ − *E*_SBNR_where *E*_MNR/SBNR heterojunction_, *E*_MNR_, and *E*_SBNR_ are the total energies of the MNR/SBNR heterojunction, MNR, and SBNR, respectively. Table 2 presents the calculated formation energies. Notably, all of these values are negative, signifying that the formation of MNR/SBNR heterojunctions is energetically favorable.

**Table 2:** Calculated formation energy *E*_form_ (meV) and total magnetization (Bohr mag per cell) along the *z*-axis of heterojunction samples

Systems	12-Ti_2_CO_2_ ZNR/12-SBZNR (conf I)	15-Ti_2_CO_2_ ZNR/15-SBZNR (conf I)	6-Zr_2_CO_2_ ANR/6-SBANR (conf I)	7-Zr_2_CO_2_ ANR/7-SBANR (conf 1)	6-Sc_2_CF_2_ ANR/6-SBANR (conf 2)	7-Sc_2_CF_2_ ANR/7-SBANR (conf 1)
*E*_form_ (meV)	−255	−431	−1107	−1259	−790	−1008
Total magnetization along the *z*-axis (Bohr mag per cell)	3.39	4.41	0.04	0.01	0.02	0.17

**Fig. 8:**
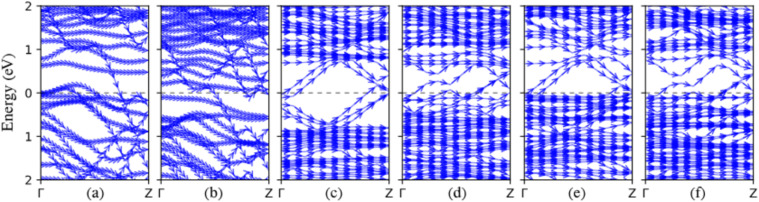
The band structure of heterojunction samples: (a) 12-Ti_2_CO_2_ ZNR/12-SBZNR, (b) 15-Ti_2_CO_2_ ZNR/15-SBZNR, (c) 6-Zr_2_CO_2_ ANR/6-SBANR, (d) 7-Zr_2_CO_2_ ANR/7-SBANR, (e) 6-Sc_2_CF_2_ ANR/6-SBANR, and (f) 7-Sc_2_CF_2_ ANR/7-SBANR. The Fermi energy is adjusted to zero.

The band structures of heterojunction systems are shown in Fig. 8. Our results reveal that all MNR/SBNR heterojunctions exhibit metallic electronic properties. The emergence of metallic behavior in the MNR/SBNR heterojunctions can be predominantly attributed to the robust interlayer interactions facilitated by van der Waals forces. These interactions play a pivotal role in promoting efficient charge transfer between the MNR and borophene layers, thereby engendering metallic properties in the composite structure. The meticulous consideration of van der Waals forces through DFT-D3 correction ensures structural stability and coherence of the heterojunction, fostering the establishment of metallic states proximal to the Fermi level. Additionally, the complementary electronic characteristics inherent to MNR and borophene contribute synergistically to the observed metallic behavior. Thus, it is the combined effect of robust interlayer interactions and intrinsic electronic properties that underpins the metallic attributes manifested in the MNR/SBNR heterojunctions. We also conducted an investigation into the magnetism of our samples, a crucial aspect of our study. Table 2 displays the total magnetization along the *z*-axis observed in our systems. Notably, all samples exhibit magnetism. Interestingly, zigzag edge configurations demonstrate higher magnetization compared to armchair edge systems, highlighting the significant impact of edge effects on magnetism. So, for a more comprehensive exploration of magnetization in the heterojunction systems, we investigated two systems: 12-Ti_2_CO_2_ ZNR/12-SBZNR and 15-Ti_2_CO_2_ ZNR/15-SBZNR. We applied electric fields of 0.2, 0.4, and 0.6 volts per angstrom in different directions and examined the noncollinear magnetization. The total magnetization of the systems was found to be insignificant in the *X* and *Y* directions, except when a 0.6 V Å^−1^ electric field was applied in the *Y* direction to the 15-Ti_2_CO_2_ ZNR/15-SBZNR system, resulting in the total magnetization of 0.92 and 0.4 Bohr magnetons per cell in the *X* and *Y* directions, respectively. However, the total magnetization in the *Z* direction was significant. When electric fields of specified amplitudes were applied to 12-Ti_2_CO_2_ ZNR/12-SBZNR in the *X*, *Y*, and *Z* directions, the total magnetization along the *Z* direction increased with 0.2 volts per angstrom electric field. Subsequently, with further increases in the electric field amplitude, the total magnetization of the system decreased. Notably, applying the electric field in the *X* direction on the 12-Ti_2_CO_2_ ZNR/12-SBZNR system resulted in insignificant changes in the increase and decrease of total magnetization. For the 15-Ti_2_CO_2_ ZNR/15-SBZNR system, it was observed that total magnetization decreased with increasing electric field, except in the *Z* direction, where applying a 0.2 V Å^−1^ field initially decreased the total magnetization, followed by an increase with a 0.4 V Å^−1^ field, and ultimately a decrease with a 0.6 V Å^−1^ field. Table 3 presents the total magnetization of 12-Ti_2_CO_2_ ZNR/12-SBZNR and 15-Ti_2_CO_2_ ZNR/15-SBZNR along the *z* direction under different amplitudes of applied electric fields in various directions. The observed significant changes in magnetization behavior upon applying electric fields can serve as a foundation for advancements in various subfields, such as multiferroics or electrically controllable antiferromagnets. Furthermore, these findings hold potential for applications in spintronic devices, as well as the development of memory devices and sensors.

**Table 3:** Calculated total magnetization along the *z*-axis (Bohr mag per cell) by applying electric fields of 0.2, 0.4, and 0.6 volts per angstrom in the *X*, *Y*, and *Z* directions

	Systems	*X*			*Y*			*Z*		
		0.2	0.4	0.6	0.2	0.4	0.6	0.2	0.4	0.6
Total magnetization along the *z*-axis (Bohr mag per cell)	12-Ti_2_CO_2_ ZNR/12-SBZNR	3.40	3.39	3.39	3.40	3.26	3.18	3.96	3.96	3.18
	15-Ti_2_CO_2_ ZNR/15-SBZNR	4.37	4.31	4.26	4.39	4.31	3.36	4.32	4.34	4.33

**Fig. 9:**
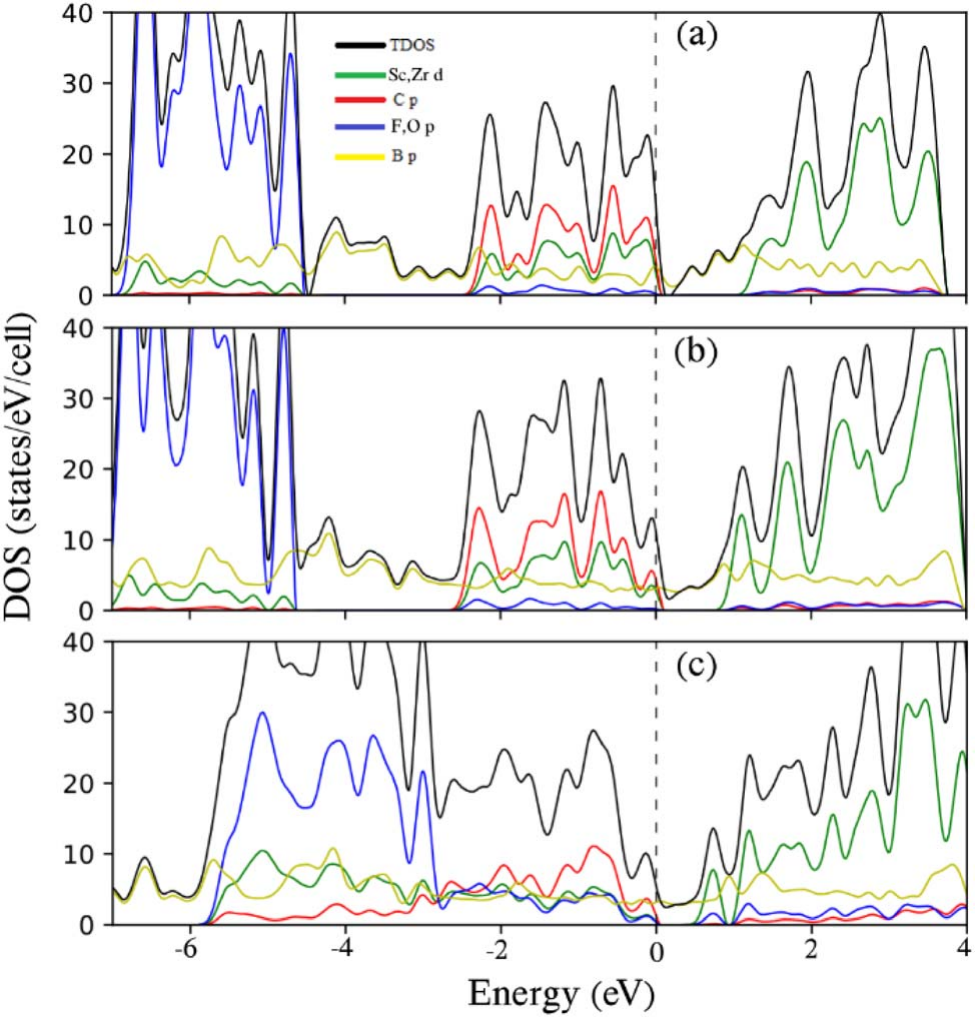
TDOS and PDOS on chosen atomic orbitals of (a) 6-Sc_2_CF_2_ ANR/6-SBANR, (b) 7-Sc_2_CF_2_ ANR/7-SBANR, and (c) 7-Zr_2_CO_2_ ANR/7-SBANR. The Fermi energy is adjusted to zero.

To provide additional insights, the DOS and PDOS for representative MNR/SBNR heterojunctions are plotted in Fig. 9. For the two types of MNR/SBNR heterojunctions, specifically for 6-Sc_2_CF_2_ ANR/6-SBANR and 7-Sc_2_CF_2_ ANR/7-SBANR, the valence states between −7 and 0 eV can be categorized into three subbands. Subband I, ranging from ∼−2.5 to 0 eV, exhibits nearly equal contributions from Sc d and C p (with more dominance from C p) orbitals. Subband II, spanning from ∼−4.5 to ∼−2.5 eV, is predominantly characterized by B p orbitals. Subband III, between −7 and ∼−4.5 eV, is primarily governed by the p orbitals of F atoms. At the Fermi energy level, there are Sc d, C p, and B p states (with C p having more dominance). Beyond the Fermi level, up to 1 eV, the bands are dominated by B p, and thereafter, the bands are characterized by the d and p orbitals of Sc and B atoms, respectively. The PDOS analysis of 6-Zr_2_CO_2_ ANR/6-SBANR indicates that from −1 to 1 (before and after the Fermi energy), predominantly, the states are dominated by B p. Between −1 and −3, there are nearly equal contributions from Zr d, C p, O p, and B p, while beyond −3 to −6, the dominance shifts to O p, and from −6 to −7, the states are entirely dominated by B p. Similar PDOS characteristics are observed for 7-Zr_2_CO_2_ ANR/7-SBANR, 12-Ti_2_CO_2_ ZNR/12-SBZNR, and 15-Ti_2_CO_2_ ZNR/15-SBZNR, with slight variations. In these cases, the energy levels between −1 and 0 demonstrate a balanced contribution from M = Ti d, Zr d, C p, O p, and B p states. As for 15-Ti_2_CO_2_ ZNR/15-SBZNR, the energy levels between 0 and 1 and −6 and −7 showcase the dominance of Ti d and B p states, respectively, along with supplementary contributions from other states.

## 5 Conclusions

In this study, we conducted a comprehensive investigation into the electronic properties of selected functionalized MXene nanoribbons (Ti_2_CO_2_, Zr_2_CO_2_, and Sc_2_CF_2_) and four types of striped borophene nanoribbons using first-principles calculations. Our findings indicate that the energy gaps of MXene nanoribbons can be tailored based on the crystallographic orientation and widths. Specifically, armchair MXene nanoribbons exhibit semiconductor behavior, while zigzag nanoribbons generally display zero band gaps, except for certain widths. The semiconductor/metallic characteristics of the nanoribbons are elucidated through an electron counting rule. Furthermore, our study reveals that all striped borophene nanoribbons inherit a metallic character from their 2D counterpart. Subsequently, computational investigations on the electronic properties of MNR/SBNR heterojunctions were carried out. The small lattice mismatch in the periodic direction and negative formation energies indicate the significant thermodynamic stability and feasibility of MNR/SBNR heterojunctions. Notably, all heterojunction samples exhibit metallic properties. Furthermore, we found significant changes in total magnetization when applying electric fields of varying directions and amplitudes to the heterojunction samples, indicating their potential utility in various applications such as multiferroics, electrically controllable antiferromagnets, spintronic devices, and memory devices and sensors.

## Data availability

The datasets generated and/or analyzed during the current study are available from the corresponding authors on reasonable request.

## Author contributions

In accordance with the Contributor Roles Taxonomy (CRediT), this delineation illustrates each author's specific contributions across various stages of manuscript development as follows: Mahdi Shirazinia, as the first author, contributed to writing – original draft and was responsible for drafting the initial manuscript. Additionally, he was engaged in software tasks, utilizing relevant software tools for data analysis and processing, and participated in the investigation phase, contributing to the acquisition of research results. Edris Faizababdi, as the corresponding author and second author, was involved in writing – review & editing, playing a crucial role in revising and refining the manuscript. He also conducted analysis, focusing on meticulous data analysis, and took charge of visualization, presenting the data and results effectively.

## Conflicts of interest

There are no conflicts to declare.
